# Validity of Wearable Inertial Sensors for Postural Sway Analysis: A Systematic Review

**DOI:** 10.3390/diagnostics16132101

**Published:** 2026-07-04

**Authors:** Giuseppe Prisco, Noemi Pisani, Maria Romano, Francesco Amato, Fabrizio Esposito, Leandro Donisi

**Affiliations:** 1 Department of Advanced Medical and Surgical Sciences, University of Campania Luigi Vanvitelli, 80138 Naples, Italy; giuseppe.prisco95@gmail.com (G.P.); noemi.pisani@unicampania.it (N.P.); fabrizio.esposito@unicampania.it (F.E.); 2 Department of Information Technology and Electrical Engineering, University of Naples Federico II, 80125 Naples, Italy; mariarom@unina.it (M.R.); framato@unina.it (F.A.)

**Keywords:** postural sway, concurrent validity, posturography, wearable sensors

## Abstract

**Background/Objectives**: Force platforms and optoelectronic motion capture systems are considered gold standards for postural sway assessment, although their use is confined to dedicated laboratory settings. Wearable inertial systems represent a practical alternative; however, their validity compared with reference systems within a shared physical domain (i.e., displacement domain) remains insufficiently investigated. This methodological requirement, frequently overlooked in the existing literature, is here adopted as an explicit inclusion criterion for the first time to ensure an appropriate metrological comparison. This review critically examines the validity of inertial systems for postural sway assessment, only including studies in which sway parameters derived from inertial measurement units (IMUs) were expressed in the same physical domain as the corresponding reference measurements. **Methods**: A systematic search of the Scopus database was conducted to identify English-language studies published up to January 2026 that compared IMU-derived sway parameters with those obtained from gold-standard systems, using parameters expressed in consistent measurement units. Sensor placement, postural tasks, signal processing techniques, extracted sway parameters, and statistical validation methods were analyzed as key methodological aspects. **Results**: Eight studies published between 2015 and 2022 met the inclusion criteria. The predominant configuration consisted of a single lumbar-mounted IMU, and quiet bipedal standing was the most frequently investigated postural task. Velocity-based parameters, particularly mean sway velocity, demonstrated moderate to high agreement with reference systems. In contrast, spatial dispersion measures, including the 95% confidence ellipse area and root mean square displacement, showed greater variability and, in some cases, systematic bias in Bland–Altman analyses. **Conclusions**: Wearable inertial systems demonstrated strong potential for estimating global and velocity-related sway parameters during quiet standing, supporting their clinical applicability. However, spatial metrics and dynamic postural tasks remain more challenging for IMU-based assessment. Methodological standardization of validation protocols and signal processing pipelines is essential to improve comparability and reproducibility across studies.

## 1. Introduction

Human postural control reflects the continuous and dynamic interaction among sensory integration, neuromuscular coordination, and biomechanical constraints [1,2,3]. During quiet standing, the human body exhibits small and continuous oscillations, known as postural sway, which reflect the activity of the postural control system [4,5]. The quantitative analysis of these oscillations is performed through posturography, which represents an established framework for balance assessment [6]. Postural sway is described using two complementary biomechanical measures: the center of pressure (CoP) and the center of mass (CoM). The CoP is defined as the point of application of the resultant ground reaction force and reflects the output of the neuromuscular system in maintaining balance and preventing falls [7,8]. The CoM represents the mass-weighted average position of all body segments in three-dimensional space and is continuously regulated by the central nervous system to maintain balance within the base of support [7,9,10]. Under quasi-static conditions such as quiet standing, the CoP and the CoM are related through a precise mechanical coupling. The CoP acts as a neuromuscular control variable, continuously oscillating around the ground projection of the CoM with larger and faster displacements in order to generate the horizontal ground reaction forces required to decelerate and redirect CoM motion [7].

Currently, the objective and reproducible quantification of postural assessment is based on computerized posturography [11]. This methodology relies on the integration of force platform and optoelectronic motion capture (OMC) systems, which represent key technologies for the simultaneous measurement of the kinetic and kinematic dynamics of the human body. Force platforms are widely regarded as the gold standard for posturographic analysis, allowing accurate estimation of the CoP trajectory during standing or movement tasks [7,12]. OMC systems provide high-precision three-dimensional kinematic data during quiet standing or dynamic postural tasks, enabling detailed reconstruction of whole-body kinematics and estimation of CoM motion [13,14,15]. Postural sway analysis based on force platform and OMC allows the extraction of multiple parameters in both the time- and frequency-domain, objectively quantifying balance performance [16,17]. These metrics are widely used in clinical diagnostics, rehabilitation monitoring, fall-risk assessment, sports performance evaluation, and ergonomics [18,19,20,21]. Although these systems ensure high analytical accuracy, their use is generally confined to controlled laboratory environments and requires trained personnel, representing a significant trade-off between measurement precision and practical applicability [22]. Consequently, these limitations restrict their adoption in routine clinical practice and hinder their application in real-world settings [23].

In recent years, inertial measurement units (IMUs) have emerged as a promising alternative for postural sway assessment [24]. IMUs integrate accelerometers, gyroscopes, and magnetometers to measure the linear accelerations and angular velocities of body segments. Their small size, portability, and ease of use enable balance assessment in real-world environments, supporting applications such as home-based monitoring, telemedicine, and long-term assessment of postural control [25]. This technological advancement is particularly relevant for clinical populations, including older adults at risk of falls, individuals with neurological disorders such as Parkinson’s disease, those with multiple sclerosis, patients with post-stroke neurological impairments, and subjects undergoing balance rehabilitation.

Several studies have investigated the use of IMUs for postural control estimation by comparing their outputs with reference systems such as force platform and OMC systems. In light of the increasing use of IMUs in clinical and real-world contexts, establishing robust evidence of their validity is essential to support their use in the assessment of postural sway. However, a critical and frequently overlooked methodological issue remains. Force platforms and OMC systems operate in the displacement domain, whereas IMUs measure inertial signals, such as linear accelerations and angular velocities, which belong to a different physical domain. Achieving domain alignment requires the implementation of dedicated strategies, including signal preprocessing, sensor fusion algorithms, biomechanical body modeling, and sensor calibration procedures. To the best of our knowledge, no previous systematic review has imposed domain alignment as an explicit inclusion criterion for validity studies of IMU-based postural sway assessment. In the absence of appropriate domain alignment, the comparison of physically non-homogeneous parameters compromises the methodological validity of concordance analyses and precludes reliable conclusions regarding measurement interchangeability. Disregarding this requirement may yield spuriously high or low agreement statistics, potentially leading researchers to over- or under-estimate the validity of IMU-based systems.

Addressing this methodological gap, the objective of this systematic review is to critically synthesize the evidence regarding the concurrent validity of wearable inertial sensors for postural sway assessment, as established through direct comparison with force platforms or OMC systems. This review only included studies in which IMU-derived sway parameters were expressed within the same physical domain as the corresponding gold-standard measurements, through the application of appropriate biomechanical models or signal processing strategies capable of converting raw inertial signals into displacement-based measures, thereby enabling direct agreement evaluation. To this end, the review specifically aimed to: (i) identify the experimental protocols and postural tasks adopted for validation; (ii) examine the sensor configurations, biomechanical models, and signal processing strategies used to estimate sway parameters from IMUs and reference systems; and (iii) assess the level of agreement reported across different sway parameters and task conditions.

## 2. Methods

### 2.1. Search Methodology

This systematic literature review was conducted in accordance with the Preferred Reporting Items for Systematic Reviews and Meta-Analyses (PRISMA) 2020 guidelines [26] (the checklist is included in the Supplementary Materials). A completed PRISMA 2020 checklist is provided as Supplementary Materials. The search was performed in the Scopus electronic database, restricting results to English-language documents published up to January 2026. Specifically, the database was queried using the following search string: ((“AGREEMENT”) OR (“BENCHMARKING”) OR (“VALIDATION”)) AND ((“FORCE PLATFORM”) OR (“OPTOELECTRONIC SYSTEM”) OR (“STEREOPHOTOGRAMMETRIC SYSTEM”)) AND ((“WEARABLE SENSOR”) OR (“IMU”) OR (“INERTIAL MEASUREMENT UNIT”) OR (“ACCELEROMETER”)) AND ((“POSTUROGRAPHY”) OR (“SWAY ANALYSIS”) OR (“STABILOMETRY”) OR (“POSTURAL CONTROL”) OR (“BALANCE”)).

### 2.2. Study Selection

Once the list of records retrieved from the Scopus database was obtained, a multi-stage screening process was conducted. First, all records were screened based on titles and abstracts to exclude studies that did not meet the predefined inclusion criteria or that satisfied any of the exclusion criteria. Articles considered potentially relevant were subsequently assessed through full-text screening to determine their eligibility.

The following general exclusion criteria were applied: studies not published in English, conference papers, review articles, book chapters, and documents not available in full-text format. In addition, the following specific exclusion criteria were established:

- studies assessing postural sway parameters without the use of wearable inertial sensors;
- studies not including agreement (concurrent validity) analyses;
- studies using wearable inertial sensors for postural sway assessment that did not include comparison with force platforms or OMC systems.

A further critical requirement was that IMU-derived sway parameters had to be expressed in the same physical domain as those obtained from the reference system. Studies reporting IMU outputs in incompatible units were excluded, as meaningful agreement analysis requires measurements within a shared domain.

The screening process was conducted in sequential stages. Following the initial database search, two independent reviewers screened titles and abstracts of all identified records for eligibility. Subsequently, the same reviewers independently performed full-text assessments of potentially relevant studies. Any discrepancies were resolved through discussion and consensus.

**Figure 1:**
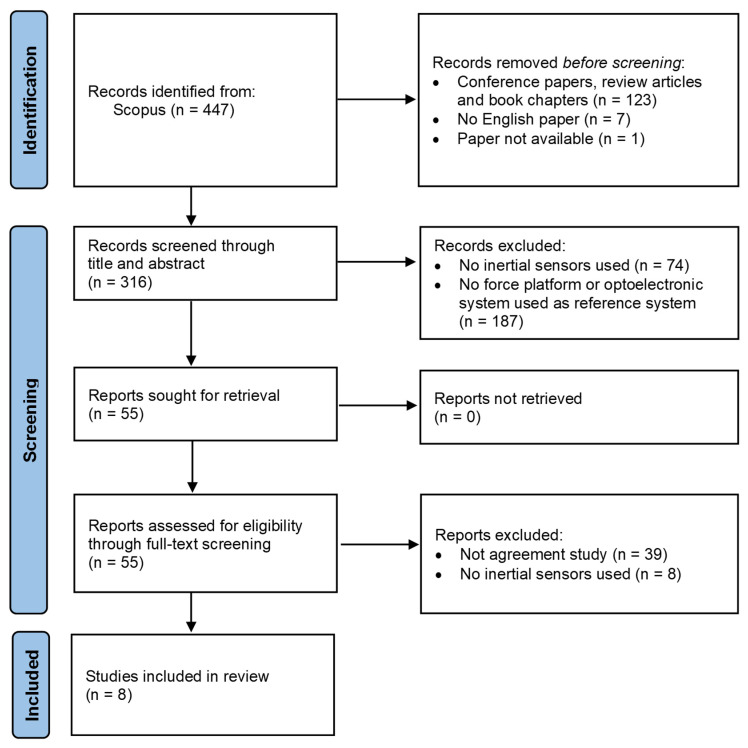
Summary review workflow.

The overall screening and selection process is summarized in the PRISMA flow diagram presented in Figure 1.

### 2.3. Risk of Bias Assessment

The methodological quality of the included studies was assessed using the Criterion Validity box of the COSMIN Risk of Bias checklist [27], originally developed for studies on patient-reported outcome measures and adapted here for agreement studies comparing wearable inertial sensors with reference systems. For each study, two standards were rated: (i) whether an appropriate correlation coefficient or area under the curve (AUC) was calculated for continuous outcome variables, and (ii) whether any other important methodological flaws affecting the validity of the reported results were identified. Following the COSMIN “worst score counts” principle, the lowest rating obtained on either standard determined the overall judgment for each study. The risk of bias assessment was conducted by two independent reviewers, and any disagreements were resolved through discussion.

## 3. Results and Discussion

**Figure 2:**
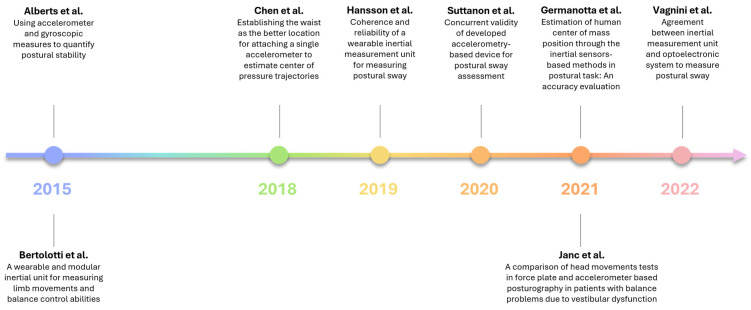
Temporal distribution of the included articles. Temporal distribution of the included articles. Alberts et al. [28], Bertolotti et al. [29], Chen et al. [30], Hansson et al. [31], Suttanon et al. [32], Germanotta et al. [33], Janc et al. [34], Vagnini et al. [35].

A total of eight articles, published between 2015 and 2022, reporting comparisons between wearable sensors and force platforms or OMC systems, were included. Although no temporal restrictions were in the search strategy, all retrieved studies were published within the last decade. Figure 2 illustrates the temporal distribution of the included articles.

Table 1 lists the included studies in chronological order based on year of publication and summarizes their characteristics according to the following criteria: study aim; participant population (including sample size and whether participants were healthy or had specific clinical conditions); type of wearable sensor used (including number of sensors, placement, and whether the device was commercial or prototype-based); algorithms used for comparison between devices; reference (gold standard) system; postural sway parameters derived from both systems; experimental protocol and tasks performed; statistical analyses used for between-system comparison; and main study findings.

### 3.1. Risk of Bias Assessment Results

Applying the Criterion Validity box of the COSMIN Risk of Bias checklist, six of the eight included studies were rated as having very good methodological quality, while two studies were rated as inadequate, for markedly different reasons (Table S1, Supplementary Materials).

Alberts et al. [28] reported a comprehensive Bland–Altman analysis and mean absolute percentage error to quantify agreement between the iPad2- and Neuro-Com-derived equilibrium scores, but did not report a correlation coefficient or AUC (the specific statistical criterion required by this COSMIN item). This illustrates a known limitation of applying a checklist developed for patient-reported outcome measures to instrumental validity studies: the item privileges the presence of a correlation coefficient over an arguably more informative agreement-based approach, which this review itself identifies as central to establishing genuine measurement equivalence. The inadequate rating for this study should therefore be interpreted as reflecting a methodological choice rather than a substantive flaw in study design.

In contrast, the inadequate rating assigned to Chen et al. [30] reflects a genuine methodological concern: the machine learning algorithms used to estimate CoP trajectories from accelerometer data were fitted without a reported independent test set or cross-validation procedure, raising the possibility that the reported correlation coefficients and error ratios reflect model fit rather than genuine predictive validity.

### 3.2. Wearable Inertial Systems and Sensor Placement

The rapid evolution of wearable technologies has been driven by continuous advances in sensor miniaturization and integration [36]. These developments have enabled the continuous monitoring of physiological and biomechanical signals, supporting a wide range of healthcare applications, including the management of chronic and degenerative conditions [37,38,39,40,41,42,43]. The studies included in this review specifically investigated wearable inertial systems and their placement on the human body for the estimation of postural sway parameters, with the aim of evaluating their performance in comparison with reference systems.

**Figure 3:**
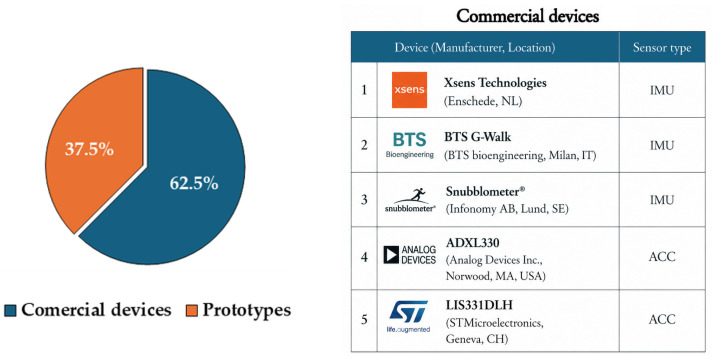
Distribution of wearable sensors: prototypes and commercial wearable inertial systems. For each commercial device, the manufacturer, location, and sensor type (IMU or accelerometer) are reported.

The analyzed devices were classified into two categories: (1) prototype systems, referring to experimental configurations not commercially available, and (2) commercial devices, defined as systems already available on the market (Figure 3).

Of the eight studies included in the review, three (37.5%) employed prototype devices. Among commercial devices, IMUs were the most commonly adopted solutions; however, two studies relied on individual sensing components, most notably accelerometers, to derive postural metrics.

**Figure 4:**
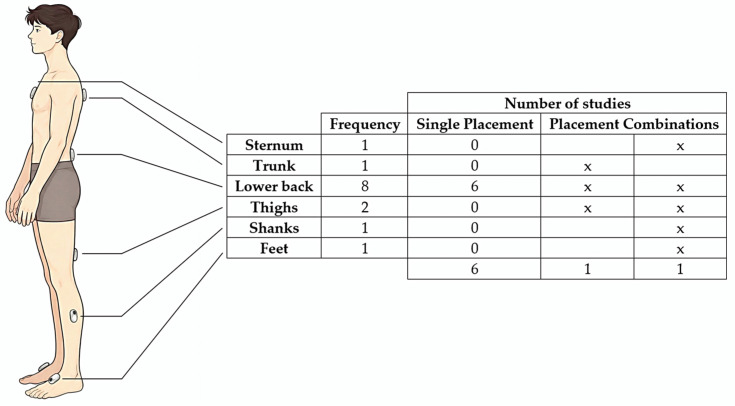
The number of studies that positioned IMUs on specific anatomical locations. The “Single Placement” column represents studies where sensors were located at only one anatomical site. The “Placement Combinations” columns represent studies where sensors were positioned at multiple anatomical locations. Each relevant location is marked with an “x”, and the number of studies utilizing that specific combination is noted at the bottom of each column. The “Frequency” reflects the cumulative number of studies that placed sensors at the respective anatomical location.

Sensor placement represents a key methodological choice in inertial-based postural assessment. IMUs can be positioned on different body segments, and multiple configurations have been explored across studies (Figure 4). All included studies placed at least one sensor at the lower back (lumbar region). Moreover, lumbar placement was the only single-sensor configuration adopted, being used in six out of eight studies. Placement of the sensor at the lumbar level approximates the location of the body’s CoM, allowing the capture of global body motion [7]. Since postural sway is commonly interpreted in terms of CoP displacement, which reflects the neuromuscular control of CoM dynamics, a sensor positioned close to the CoM can effectively characterize whole-body oscillations during quiet standing [44,45].

Two studies implemented multi-sensor configurations. Chen et al. [30] investigated the optimal placement of a single accelerometer for estimating CoP trajectories during quiet standing. Three configurations were compared, with sensors positioned on the upper trunk, lower back (L2–L3 level), and lower thigh. Accelerometer signals were processed using supervised machine learning algorithms to estimate CoP displacement and were validated against force platform measurements. The authors reported that the lower back configuration yielded lower error ratios (ER) and higher correlations with reference CoP trajectories compared to trunk and thigh placements. In particular, anterior–posterior (AP) estimates were most accurate at the lumbar level, supporting the hypothesis that sensor placement close to CoM enhances the reconstruction of global postural sway. The study concluded that a single waist-mounted sensor provides an optimal balance between simplicity and accuracy for CoP estimation during quiet stance. In contrast, Germanotta et al. [33] evaluated two IMU-based approaches for estimating whole-body CoM displacement during postural tasks of increasing difficulty. The first approach employed a single IMU placed on the pelvis and applied a strapdown integration (SDI) method, estimating CoM displacement through double integration of acceleration signals. The second approach relied on a multi-sensor biomechanical model (BM) incorporating seven IMUs positioned on the pelvis and lower limbs. Both configurations were validated against an OMC system. The results indicated that the SDI single-sensor approach achieved acceptable accuracy during tasks characterized by small-amplitude oscillations (e.g., double-leg stance). However, the BM approach significantly outperformed the single-sensor method in tasks involving larger CoM excursions (e.g., sway and squat tasks). The distributed sensor network enabled more accurate reconstruction of segmental kinematics and reduced integration drift, resulting in improved agreement with the reference system. Collectively, these findings highlight a fundamental trade-off in inertial system design. Single-sensor approaches, particularly when positioned at the lumbar level, are practical, unobtrusive, and sufficiently accurate for low-amplitude postural sway assessment during quiet standing. However, as task complexity increases or when detailed analysis of segmental contributions to balance control is required, multi-sensor configurations provide superior biomechanical fidelity and robustness. An additional factor influencing system performance is the computational strategy employed. SDI methods are inherently susceptible to drift accumulation, particularly in static or quasi-static conditions where zero-velocity updates cannot be reliably applied. In contrast, biomechanical modeling approaches distribute estimation across multiple body segments, mitigating drift effects and improving spatial reconstruction accuracy.

Therefore, although single-sensor placement at the lumbar level remains the most commonly adopted configuration in postural analysis, current evidence suggests that multi-sensor systems may be preferable in applications requiring higher accuracy, dynamic task evaluation, or detailed segmental analysis.

### 3.3. Postural Sway Tasks

Across all included studies, participants performed postural tasks while simultaneously using wearable inertial sensors and a reference system, namely a force platform or an OMC system. When reported, trial duration ranged from 20 to 60 s, and several protocols included repeated trials separated by rest intervals to improve measurement reliability. Despite differences in terminology and procedural details, the experimental paradigms adopted across studies can be grouped into three main categories: (i) structured sensory manipulation protocols, (ii) static quiet standing tasks, and (iii) voluntary or dynamic postural tasks (Table 2).

A structured sensory rebalancing paradigm was implemented by Alberts et al. [28] through the Sensory Organization Test (SOT), consisting of six 20-s conditions combining visual manipulations (eyes-open (EO), eyes-closed (EC), sway-referenced vision) with variations in support surface (static or dynamic platform). This protocol systematically challenges the integration of visual, vestibular, and somatosensory inputs involved in postural control and enables the evaluation of adaptive balance strategies under progressively destabilizing conditions. Two additional studies by Bertolotti et al. [29] and Janc et al. [34] adopted the modified Clinical Test of Sensory Interaction on Balance (mCTSIB), which includes four quiet-standing conditions performed with EO and EC on both firm and compliant (foam) surfaces. Although the experimental procedure of the mCTSIB was consistent across these studies, differences in trial duration and repetition schemes were observed, highlighting procedural heterogeneity even within nominally standardized protocols.

Quiet standing on a firm surface under EO and EC conditions represented the most frequently used paradigm and was either assessed independently or embedded within structured sensory protocols. Hansson et al. [31] examined postural sway during 30-s trials in both EO and EC conditions, instructing participants to fixate a target during EO trials to standardize the visual input. Vagnini et al. [35] extended static assessment by including both double-leg stance and single-leg stance on the left and right limbs under EO and EC, each lasting approximately 60 s. The single-leg condition reduced the base of support and increased postural demand, resulting in larger CoM excursions compared to bipedal stance.

Beyond static paradigms, several studies introduced voluntary sway or dynamic tasks to further challenge postural control and evaluate inertial sensor performance under larger or intentional CoM displacements. Chen et al. [30] required participants to perform controlled AP and mediolateral (ML) sway excursions lasting approximately 40 s. Similarly, Germanotta et al. [33] combined double-leg and single-leg stance conditions with AP and ML sway tasks and further included free pelvic sway and squat movements, thereby increasing biomechanical complexity and segmental involvement beyond conventional static posturography. In a different approach, Suttanon et al. [32] implemented directional leaning tasks (forward, backward, left, and right) under EO conditions, effectively assessing limits of stability rather than quiet standing. These tasks required participants to intentionally displace their CoM toward the boundaries of the base of support, imposing greater demands on balance regulation mechanisms.

Overall, although quiet standing on a firm surface under EO and EC conditions was common in most validation studies, considerable methodological heterogeneity was observed in sensory manipulation, surface compliance, base of support configuration, inclusion of voluntary sway or functional movements, and trial duration. Given the task-dependent nature of postural sway parameters, this variability is likely to influence the magnitude and characteristics of sway patterns, helping to explain, at least in part, the discrepancies in the reported agreement between wearable inertial sensors and reference systems.

### 3.4. Signal Processing and Kinematic Reconstruction

The conversion of raw inertial signals into postural sway parameters expressed in the same physical domain as reference systems represents one of the most critical methodological steps in IMU-based postural sway analysis. Across the studies included in this review, substantial heterogeneity emerged in both signal pre-processing procedures and body kinematic reconstruction strategies used to estimate CoM- or CoP-related metrics. Table 3 summarizes the signal pre-processing and sway estimation methods adopted in each study.

Three of the eight studies did not report details on signal pre-processing [31,32,33]. Bertolotti et al. [29] applied a 0.4 Hz low-pass filter to acceleration signals and used the filtered signal as a static inclinometer within an inverted pendulum model. Janc et al. [34] combined low-pass filtering with down-sampling to 20 Hz to meet the requirements of the Madgwick orientation algorithm, while Chen et al. [30] performed filtering and down-sampling prior to training machine learning models for CoP estimation. More structured pipelines were described by Alberts et al. [28], who applied a fourth-order Butterworth low-pass filter at 1.25 Hz and corrected sensor orientation offsets prior to nonlinear modeling, and by Vagnini et al. [35], who used a sixth-order Butterworth filter at 5 Hz with linear detrending to reduce baseline drift.

Beyond pre-processing, studies adopted different kinematic reconstruction strategies to derive sway-related parameters from inertial data. Hansson et al. [31] extracted mean sway velocity (MVELO, MVELO_AP_, MVELO_ML_) directly from a commercial IMU without reporting internal processing details. Janc et al. [34] computed angular sway velocity from trunk orientation quaternions estimated via the Madgwick algorithm, using orientation changes as a proxy for postural sway. Germanotta et al. [33] estimated CoM displacement through double integration of pelvis accelerations, using the SDI approach.

Other studies relied on geometric approaches to estimate sway parameters. Suttanon et al. [32] derived CoM sway angles from acceleration signals using arctangent-based trigonometric equations and applied a Kalman filter to reduce angular noise. Vagnini et al. [35] reconstructed displacement from tri-axial acceleration signal using trigonometric projections and sensor height as a geometric reference. These methods reduce the cumulative drift associated with double integration but rely on simplified assumptions regarding body geometry and rigid-body behavior.

Data-driven approaches replaced explicit physical modeling with empirical mappings calibrated against reference systems. Chen et al. [30] trained neural networks (NN), genetic algorithms (GA), and adaptive network-based fuzzy inference systems (ANFIS) to predict CoP trajectories from acceleration signals recorded at different anatomical locations. Alberts et al. [28] developed a nonlinear mixed-effects model combining a five-knot restricted cubic spline with a sine function to obtain the equilibrium score parameter from inertial signals. Although these methods can capture nonlinear relationships between trunk motion and ground reaction forces, they require calibration against a gold standard and may exhibit limited generalizability across populations or task conditions.

Finally, two of the eight studies employed physics-based biomechanical models. Bertolotti et al. [29] used a single-link inverted pendulum model, projecting trunk pitch and roll onto the ground plane using sensor height to estimate horizontal CoM displacement. Germanotta et al. [33] implemented a multi-segment biomechanical model based on seven IMUs placed on the pelvis and lower limbs. Kalman-filtered segment orientations were integrated into a kinematic chain to reconstruct whole-body CoM dynamics.

### 3.5. Postural Sway Parameters

Sway parameters are essential metrics that characterize human balance. Table 4 groups postural sway parameters into four main categories: (i) spatial parameters, (ii) speed parameters, (iii) stability indices, and (iv) raw trajectories.

Spatial parameters describe the extent of body sway in terms of displacement, area, or excursion. Among these, the root mean square of the distance (RMS-DIST) from the mean CoP or CoM position was used to quantify overall sway amplitude by Bertolotti et al. [29], while Vagnini et al. [35] analyzed the RMS-DIST in AP and ML directions (RMS-DIST_AP_/RMS-DIST_ML_). The 95% confidence ellipse area (CEA) was implemented in three studies [29,33,35] to characterize the two-dimensional distribution of sway points. Other spatial metrics included total path length (TOTEX) and directional excursion (TOTEX_AP_/TOTEX_ML_, RDIST_AP_/RDIST_ML_), which provide cumulative or peak-to-peak displacement measures of the CoM or CoP [33,35]. These parameters are particularly sensitive to task difficulty, base-of-support alterations, and visual or somatosensory manipulations.

Velocity parameters capture the speed of body sway and provide complementary information to spatial measures. MVELO, MVELO_AP_, and MVELO_ML_ quantify the rate of CoP or CoM displacement over time [29,31,34,35], reflecting neuromuscular responsiveness and control efficiency. Janc et al. [34] focused on angular sway velocity, defined as the change in the CoM angle per unit time, which offers an alternative method to assess dynamic postural adjustments.

Alberts et al. [28] calculated the Equilibrium Score as a stability index for the overall assessment of postural control. Finally, Chen et al. [30] did not extract sway parameters directly, instead using raw signals as input to machine learning algorithms.

Across the eight studies, a notable heterogeneity emerged in both the selection and implementation of sway parameters. Single-sensor configurations, typically located at the lower back, tended to focus on global measures such as TOTEX, RMS-DIST, or MVELO, whereas multi-sensor approaches enabled more detailed spatial decomposition (AP/ML) [33]. These methodological differences highlight the influence of sensor placement, task type, and processing strategy on the selection and interpretability of sway metrics.

### 3.6. Validation Tools

The validation tools employed across the included studies can be grouped into five categories: correlation analysis, Bland–Altman (BA) analysis, error metrics, statistical tests, and Passing–Bablok (PB) regression.

**Figure 5:**
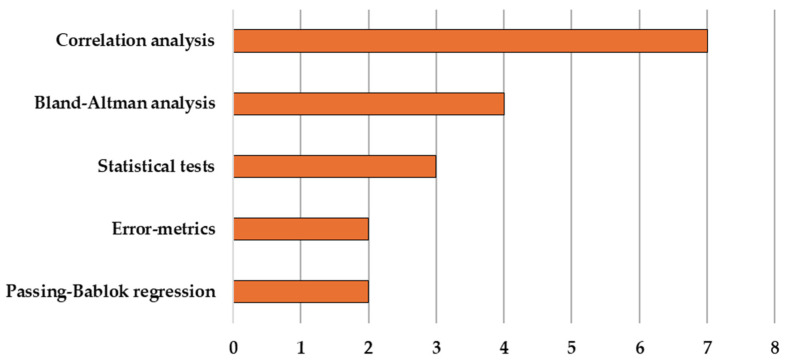
Distribution of the employed validation tools.

As illustrated in Figure 5, correlation analysis was the most frequently adopted approach, appearing in seven studies followed by the Pearson correlation coefficient (PCC) [29,30,31,32,33], used to quantify the linear association between IMU-derived and reference-system-derived parameters. The Spearman correlation coefficient (SCC) was adopted by Janc et al. [34] for non-parametric association analysis, while the intraclass correlation coefficient (ICC) was additionally reported by Vagnini et al. [35] to assess relative reliability and consistency between systems. BA analysis represented the second most frequently used approach [28,31,34,35], providing estimates of systematic bias and limits of agreement between measurement systems, and thereby offering direct evidence of potential interchangeability. Statistical tests were employed in three of the eight studies [31,33,34]. Hansson et al. [31] used paired t-test, whereas Janc et al. [34] applied the Wilcoxon signed-rank test; in both studies, inferential statistics were used to complement agreement analyses by identifying condition-specific differences between systems. Error metrics were adopted in two studies [28,30]. Alberts et al. [28] calculated the mean absolute percentage error, reporting relatively low error values across most postural conditions, while Chen et al. [30] used the error ratio (ER) to quantify the reconstruction accuracy of machine-learning models estimating CoP trajectories from accelerometer signals, reporting correlation coefficients exceeding 0.80 and ER values below 15%. PB regression was reported in two studies [29,32], selected for its robustness to outliers and suitability for method-comparison analyses that do not assume normally distributed measurement errors.

While each metric provides complementary information, none is sufficient on its own to comprehensively characterize IMU validity. Therefore, the combined use of these approaches is essential for a rigorous and interpretable validity assessment of wearable inertial systems in posturographic applications.

## 4. Conclusions

The systematic literature review underscores the potential of IMUs for estimating postural control parameters through direct comparison with traditional laboratory systems, often supported by biomechanical models to standardize the physical domain of assessment. The included studies, published between 2015 and 2022, evaluated the validity of wearable inertial systems through comparison with force platforms and OMC systems, considered gold standards in posturography. The analysis of sensor configurations showed that lumbar placement represents the most frequently adopted solution, particularly in single-sensor configurations aimed at estimating global sway parameters during quiet standing. IMUs placed near the CoM tended to provide more accurate estimates of velocity-related sway parameters, which showed moderate or high levels of agreement compared to reference systems. Conversely, spatial parameters exhibited greater variability and less consistent levels of agreement, particularly in more complex tasks requiring accurate kinematic reconstruction of the CoP or CoM trajectories. Furthermore, multi-sensor configurations and multi-segmental biomechanical models demonstrated superior performance compared with single IMU-based approaches in dynamic tasks or conditions characterized by large postural oscillations. Overall, velocity-related parameters showed more robust validity than spatial metrics.

The certainty of the evidence supporting the present findings should be considered low to moderate, reflecting several converging factors: the limited number of available studies, the methodological heterogeneity across validation protocols, and the restricted representativeness of the study populations. Despite these promising findings, several important limitations must be acknowledged. First, the evidence base remains limited, as only eight studies were included, all involving healthy participants, which substantially restricts the generalizability of the findings to clinical populations, such as older adults or individuals with neurological disorders, where IMU-based posturography would have the greatest clinical impact. Second, the substantial methodological heterogeneity across studies—including differences in sensor placement, signal pre-processing pipelines, biomechanical modeling strategies, postural tasks, and validation metrics—limits direct comparability and weakens the overall strength of the conclusions.

In conclusion, this review supports the promising role of IMUs as valid tools for assessing postural sway in healthy individuals, particularly in contexts where portability, rapid use, and easy implementation are priorities. However, because all included studies were conducted in healthy adults, the available evidence does not support the validity of IMU-based systems in Parkinson’s disease, multiple sclerosis, stroke, vestibular disorders, and other balance disorders. Consequently, conclusions regarding clinical applicability should be interpreted with caution, and dedicated validation studies in these populations should be considered a priority for future research. Although laboratory-based instrumentation remains the reference standard for high-precision biomechanical quantification, inertial systems may represent a viable alternative for monitoring and evaluating postural balance in more ecological settings. By enforcing physical domain alignment as an explicit inclusion criterion, this review provides a more metrologically robust synthesis of the available evidence than previous reviews on IMU-based postural sway validation. Future research should focus on the standardization of validation protocols and computational procedures to better define IMU performance and clarify the contexts in which these technologies can reliably complement or replace traditional posturography technologies.

## Figures and Tables

**Table 1:** Analysis of the studies included in the review.

Study	Scope	Population	Sensor Type (Number/ Positioning)	Gold-Standard System	Algorithm	Postural Sway Parameters	Postural Task	Validation Tool	Results
Alberts et al. (2015) [28]	Evaluating postural stability accuracy using iPad 2s on the lower back by comparing CoG AP sway data to NeuroCom SOT equilibrium scores	49 healthy subjects	Accelerometer: LIS331DLH, STMicroelectronics, Geneve, CH (1/lower back)	Force platform: Static Balance Master, NeuroCom International Inc., Clackamas, OR, USA	Mathematical model combining a five-knot restricted cubic spline and a sine function	Equilibrium score [%]	EO-Firm EC-Firm SRV-Firm EO-SR EC-SR SRV-SR	BA, MAPE	The two measurement systems showed good agreement with bias between 0.01% (SOT-1) and 6.2% (SOT-5) and MAPE ranging from 5.87% (SOT-5) to 10.42% (SOT-2)
Bertolotti et al. (2015) [29]	Validating a prototype IMU on the lower back for balance stability by comparing its measurements to the gold standard force platform, the Nintendo Wii Balance Board	10 healthy subjects	IMU Prototype (1/lower back)	Force platform: Wii Balance Board, Nintendo, Kyoto, Japan	Inverted pendulum model	RMS-DIST [mm], CEA [mm^2^], MVELO [mm/s]	EO-Firm EC-Firm EO-Foam EC-Foam	PCC, PB	The prototype demonstrated PCC ranging from moderate (0.598 for EO-Firm) to strong (0.933 for EC-Foam), confirming its validity for balance assessment
Chen et al. (2018) [30]	Evaluating accelerometer placement for CoP estimation during balance tasks using three machine-learning algorithms, with results compared to the gold standard force platform measurements	10 healthy subjects	Accelerometer: ADXL330, Analog Devices Inc., Norwood, MA, USA (3/trunk, lower back and thigh)	Force platform: Mems Technology Corp., New Taipei City, Taiwan	Three machine-learning algorithms for CoP estimation: NN GA ANFIS	CoP trajectory [cm]	AP sway excursion ML sway excursion	PCC, ER	The combined configurations and machine-learning algorithms achieved ER < 15% and PCC > 0.80. The lower back placement and GA showed optimal performance with 7.6% ER and 0.96 PCC
Hansson et al. (2019) [31]	Testing the validity of an IMU sensor compared with the gold standard force platform for measuring postural sway	32 healthy subjects	IMU: Snubblometer^®^, Infonomy AB, Lund, Sweden (1/lower back)	Force platform: Good Balance™, Metitur Ltd., Jyväskylä, Finland	NS	MVELO_AP_ [mm/s] MVELO_ML_ [mm/s]	EO-Firm EC-Firm	Paired t-test, BA, PCC	High correlation (0.71–0.88) was found between the two measurements, but BA analysis revealed systematic bias and poor agreement, with bias ranging from −0.93 to −3.20
Suttanon et al. (2020) [32]	Determining the correlation between an accelerometry-based device and an OMC system to evaluate accelerometer accuracy in postural sway assessment	20 healthy subjects	Accelerometer: Prototype (1/lower back)	OMC system: Vicon MX 512 M, Vicon Motion Systems, Oxford, UK	Mathematical model based on Kalman filter and trigonometric equations	CoM angle_AP_ [deg] CoM angle_ML_ [deg]	EO-Firm EO-LB EO-LF EO-LL EO-RL	PCC, PB	High correlation between OMC analysis and accelerometer prototype in AP (0.982) and ML (0.835) directions was found
Germanotta et al. (2021) [33]	Comparing the SDI approach based on a single sensor and a seven-IMU network (BM) compared with an OMC system to determine the best method for measuring CoM dynamics in postural tasks	15 healthy subjects	IMU: MTw, Xsens Technologies, Enschede, The Netherlands (8/sternum, lower back, feet, shanks and thighs)	OMC system: SMART D500, BTS bioengineering, Milan, Italy	BM and SDI approaches	RDISTAP [mm] RDISTML [mm] TOTEXAP [mm] TOTEXML [mm] CEA [cm^2^] MVELOAP [mm/s] MVELOML [mm/s]	DL-EO SL-EO AP sway excursion ML sway excursion Free-EO	PCC	The results suggest BM is preferable over SDI for accuracy, as SDI showed lower RMSE values in the AP component (6.1–32.0), but BM performed better in other tasks and exhibited stronger correlations (PCC > 0.98)
Janc et al. (2021) [34]	Comparing the results of head shaking posturography performed in accordance with the same task protocol on an IMU device and a standard force platform	65 healthy subjects	IMU: Prototype (1/lower back)	Force platform: Static Balance Master, NeuroCom International Inc., Clackamas, OR, USA	Orientation estimation using Madgwick quaternion algorithm and inverted pendulum model	Angular sway velocity [deg/s]	EO-Firm EC-Firm EO-Foam EC-Foam	Wilcoxon test, BA, SCC	The IMU exhibited a significant difference in the EO-Firm and EC-Firm, but no difference in the EO-Foam and EC-Foam, with SCC results ranging from moderate to strong (0.60 to 0.98). BA showed good agreement between the two methods
Vagnini et al. (2022) [35]	Evaluating the agreement between an IMU and an OMC system in the measurement of postural sway	15 healthy subjects	IMU: BTS G-Walk, BTS bioengineering, Milan, Italy (1/lower back)	OMC system: Smart-DX, BTS bioengineering, Milan, Italy	Inverted pendulum model	TOTEX_AP_ [mm] TOTEX_ML_ [mm] TOTEX [mm] RDIST_AP_ [mm] RDIST_ML_ [mm] RMS-DIST_AP_ [mm] RMS-DIST_ML_ [mm] MVELO_AP_ [mm/s] MVELO_ML_ [mm/s] CEA [mm^2^]	DL-EO DL-EC MS-L MS-R	ICC, BA, Mann–Whitney test	Excellent-to-good agreement of the IMU for length of TOTEX and MVELO (ICC > 0.9), disagreement for other measures (ICC < 0.75)

Abbreviations: ANFIS = adaptive network-based fuzzy inference system; AP = anterior–posterior; BA = Bland–Altman; BM = multi-sensor biomechanical model; CEA = 95% confidence ellipse area; CoG = center of gravity; CoM = center of mass; CoP = center of pressure; DL-EC = double leg—eyes closed; DL-EO = double leg—eyes open; EC-Firm = eyes closed—firm platform; EC-Foam = eyes closed—foam platform; EC-SR = eyes closed—sway-referenced platform; EO-Firm = eyes open—firm platform; EO-Foam = eyes open—foam platform; EO-LB = eyes open—leaning backward; EO-LF = eyes open—leaning forward; EO-LL = eyes open—left leaning; EO-RL = eyes open—right leaning; EO-SR = eyes open—sway-referenced platform; ER = error ratio; Free-EO = free sway—eyes open; GA = genetic algorithm; ICC = intraclass correlation coefficient; IMU = inertial measurement unit; MAPE = mean absolute percentage error; ML = mediolateral; MS-L = monopodalic stance on the left leg; MS-R = monopodalic stance on the right leg; mCTSIB = Modified Clinical Test of Sensory Interaction on Balance; MVELO = mean sway velocity; NN = neural network; NS = not specified; OMC = optoelectronic motion capture; PB = Passing–Bablok; PCC = Pearson correlation coefficient; RMSE = root mean square error; RMS-DIST = root mean square of the resultant distance; RDIST = range of the resultant distance; SCC = Spearman correlation coefficient; SDI = strapdown integration; SL-EO = single leg—eyes open; SOT = Sensory Organization Test; SRV-Firm = sway-referenced visual surround—firm platform; SRV-SR = sway-referenced visual surround—sway-referenced platform; TOTEX = total path length.

**Table 2:** Overview of postural sway assessment protocols across included studies, detailing the type of task, specific postural conditions, and trial durations.

Study	Protocol Type	Postural Tasks	Number of Tasks and Duration
Alberts et al. [28]	SOT	EO-Firm, EC-Firm, SRV-Firm, EO-SR, EC-SR, SRV-SR	6 × 20 s
Bertolotti et al. [29]	mCTSIB	EO-Firm, EC-Firm, EO-Foam, EC-Foam	4 × 40 s
Chen et al. [30]	Voluntary sway	AP sway excursion, ML sway excursion	2 × 40 s
Hansson et al. [31]	Quiet standing	EO-Firm, EC-Firm	2 × 30 s
Suttanon et al. [32]	Quiet standing & directional leaning	EO-Firm, EO-LB, EO-LF, EO-LL, EO-RL	Not specified
Germanotta et al. [33]	Mixed static & dynamic tasks	DL-EO, SL-EO, Free-EO, AP sway excursion, ML sway excursion	Not specified
Janc et al. [34]	mCTSIB	EO-Firm, EC-Firm, EO-Foam, EC-Foam	4 × 30 s
Vagnini et al. [35]	Static stance	DL-EO, DL-EC, MS-L, MS-R	4 × 60 s

Abbreviations: AP = anterior–posterior; DL-EC = double leg—eyes closed; DL-EO = double leg—eyes open; EC-Firm = eyes closed—firm platform; EC-Foam = eyes closed—foam platform; EC-SR = eyes closed—sway-referenced platform; EO-Firm = eyes open—firm platform; EO-Foam = eyes open—foam platform; EO-LB = eyes open—leaning backward; EO-LF = eyes open—leaning forward; EO-LL = eyes open—left leaning; EO-RL = eyes open—right leaning; EO-SR = eyes open—sway-referenced platform; Free-EO = free sway—eyes open; ML = mediolateral; MS-L = monopodalic stance on the left leg; MS-R = monopodalic stance on the right leg; mCTSIB = Modified Clinical Test of Sensory Interaction on Balance; SL-EO = single leg—eyes open; SOT = Sensory Organization Test; SRV-Firm = sway-referenced visual surround—firm platform; SRV-SR = sway-referenced visual surround—sway-referenced platform.

**Table 3:** Signal conditioning and sway estimation approach adopted in each included study.

Study	Signal Pre-Processing	Estimation Approach
Alberts et al. [28]	4th-order LP Butterworth filter + sensor orientation offset correction	Nonlinear mixed-effects model (5-knot restricted cubic spline + sine function)
Bertolotti et al. [29]	LP filter	Pitch/roll angles projected onto ground via inverted pendulum geometry to estimate CoM displacement
Chen et al. [30]	LP filter + downsampling	NN, GA, and ANFIS algorithms trained to predict CoP trajectory from accelerometer signals
Hansson et al. [31]	Not reported	Not reported
Suttanon et al. [32]	Not reported	Kalman filter applied to reduce angular error and arctan-based trigonometric equations
Germanotta et al. [33]	Not reported	SDI: Double integration of pelvis accelerometer signal to estimate CoM displacement BM: Kalman-filtered segment orientations to reconstruct whole-body CoM dynamics
Janc et al. [34]	LP filter + downsampling	Madgwick quaternion algorithm estimates orientation
Vagnini et al. [35]	6th-order LP Butterworth filter + linear detrending	Displacement computed from 3-axis accelerometer via trigonometric approach

Abbreviations: ANFIS = adaptive network-based fuzzy inference system; BM = multi-sensor biomechanical model; CoM = center of mass; CoP = center of pressure; GA = genetic algorithm; LP = low-pass; NN = neural network; SDI = strapdown integration.

**Table 4:** Postural sway parameters computed in the studies included in this systematic review. The parameters are grouped based on their type, with the total number of studies measuring each parameter indicated in parentheses. The table provides the “Definition” of each parameter, the “Total” number of studies reporting it, and the corresponding “Articles” where the measure was used.

Spatial sway parameter (7)			
Parameter	Definition	Total	Article
RMS-DIST	Root mean square distance of CoP/CoM from its mean value	1	[29]
RMS-DIST_AP_ RMS-DIST_ML_	Root mean square distance of CoP/CoM from its mean value in AP/ML directions	1	[35]
CEA	Area of the ellipse that contains 95% of the points on the AP and ML CoP/CoM trajectories	3	[29,33,35]
CoM angle_AP_ CoM angle_ML_	Angle of oscillation calculated between the vertical and the line connecting the ankle joint to the CoM in AP/ML directions	1	[32]
RDIST_AP_ RDIST_ML_	Excursion between maximum and minimum points of CoM in AP/ML directions	2	[33,35]
TOTEX	Total length of CoP/CoM trajectory	1	[35]
TOTEX_AP_ TOTEX_ML_	Length of CoP/CoM trajectory summing consecutive position changes in AP/ML directions	2	[33,35]
Velocity Parameters (3)			
Parameter	Definition	Total	Article
MVELO	Total length of CoP/CoM trajectory divided by duration of test	1	[29]
MVELO_AP_ MVELO_ML_	Mean velocity of CoP/CoM in AP and ML directions	3	[31,33,35]
Angular Sway velocity	Change in CoP/CoM oscillation angle divided by duration of test	1	[34]
Stability Index (1)			
Parameter	Definition	Total	Article
Equilibrium Score	Average deviation of CoG for each test in each condition	1	[28]
Raw Signals/Trajectories (1)			
Parameter	Definition	Total	Article
CoP trajectory CoM trajectory	Raw path of the CoP/CoM	1	[30]

Abbreviations: AP = anterior–posterior; CEA = 95% confidence ellipse area; CoG = center of gravity; CoM = center of mass; CoP = center of pressure; ML = mediolateral; MVELO = mean sway velocity; RMS-DIST = root mean square of the resultant distance; RDIST = range of the resultant distance; TOTEX = total path length.

## Data Availability

No new data were created or analyzed in this study. Data sharing is not applicable to this article.

## Supplementary Materials

The following supporting information can be downloaded at: https://www.mdpi.com/article/10.3390/diagnostics16132101/s1, Table S1: COSMIN Risk of Bias assessment (Criterion Validity box) of the included studies; File S1: PRISMA 2020 Checklist.

## Abbreviations

The following abbreviations are used in this manuscript:

ANFIS	Adaptive network-based fuzzy inference system
AP	Anterior–posterior
AUC	Area under curve
BA	Bland–Altman
BM	Multi-sensor biomechanical model
CEA	95% confidence ellipse area
CoG	Center of gravity
CoM	Center of mass
CoP	Center of pressure
DL-EC	Double leg—eyes closed
DL-EO	Double leg—eyes open
EC	Eyes closed
EC-Firm	Eyes closed—firm platform
EC-Foam	Eyes closed—foam platform
EC-SR	Eyes closed—sway-referenced platform
EO	Eyes open
EO-Firm	Eyes open—firm platform
EO-Foam	Eyes open—foam platform
EO-LB	Eyes open—leaning backward
EO-LF	Eyes open—leaning forward
EO-LL	Eyes open—left leaning
EO-RL	Eyes open—right leaning
EO-SR	Eyes open—sway-referenced platform
ER	Error ratio
Free-EO	Free sway—eyes open
GA	Genetic algorithm
ICC	Intraclass correlation coefficient
IMU	Inertial measurement unit
LP	Low-pass
MAPE	Mean absolute percentage error
mCTSIB	Modified Clinical Test of Sensory Interaction on Balance
ML	Mediolateral
MS-L	Monopodalic stance on the left leg
MS-R	Monopodalic stance on the right leg
MVELO	Mean sway velocity
NN	Neural network
NS	Not specified
OMC	Optoelectronic motion capture
PB	Passing–Bablok
PCC	Pearson correlation coefficient
PRISMA	Preferred Reporting Items for Systematic Reviews and Meta-Analyses
RDIST	Range of the resultant distance
RMS-DIST	Root mean square of the resultant distance
RMSE	Root mean square error
SCC	Spearman correlation coefficient
SDI	Strapdown integration
SL-EO	Single leg—eyes open
SOT	Sensory Organization Test
SRV-Firm	Sway-referenced visual surround—firm platform
SRV-SR	Sway-referenced visual surround—sway-referenced platform
TOTEX	Total path length
