# Supplementary material for: Validity of Wearable Inertial Sensors for Postural Sway Analysis: A Systematic Review
Source: Diagnostics (Basel). 2026 Jul 4;16(13):2101. doi: 10.3390/diagnostics16132101 (PMC13360212; doi:10.3390/diagnostics16132101)
Supplement: Supplementary file 1 [file diagnostics-16-02101-s001.zip › diagnostics-4368574-supplementary.pdf]

Table S1. COSMIN Risk of Bias assessment (Criterion Validity box) of the included studies.

Study	Statistical Method (Correlation/AUC)	Other Methodological Flaws	Overall Judgment
Alberts et al. (2015) [28]	Not met — only BA analysis and MAPE reported; no correlation coefficient or AUC	None identified	Inadequate
Bertolotti et al. (2015) [29]	Met — PCC	None identified	Very good
Chen et al. (2018) [30]	Met — PCC	ML models (NN, GA, ANFIS) fitted without a reported independent test set or cross-validation	Inadequate
Hansson et al. (2019) [31]	Met — PCC	None identified	Very good
Suttanon et al. (2020) [32]	Met — PCC	None identified	Very good
Germanotta et al. (2021) [33]	Met — PCC	None identified	Very good
Janc et al. (2021) [34]	Met — SCC	None identified	Very good
Vagnini et al. (2022) [35]	Met — ICC	None identified	Very good

Abbreviations: ANFIS = adaptive network-based fuzzy inference system; BA = Bland–Altman; GA = genetic algorithm; ICC = intraclass correlation coefficient; MAPE = mean absolute percentage error; ML = machine learning; NN = neural network; PCC = Pearson correlation coefficient; SCC = Spearman correlation coefficient.
